# Maternal Low Quality Protein Diet Alters Plasma Amino Acid Concentrations of Weaning Rats

**DOI:** 10.3390/nu7125508

**Published:** 2015-12-01

**Authors:** Arzu Kabasakal Cetin, Halil Dasgin, Atila Gülec, İlyas Onbasilar, Asli Akyol

**Affiliations:** 1 Department of Nutrition and Dietetics, Hacettepe University, Sıhhiye, 06100 Ankara, Turkey; arzu.kabasakal@hacettepe.edu.tr (A.K.C.); guleca@hacettepe.edu.tr (A.G.); 2 Department of Nutrition and Dietetics, Kirikkale University, Merkez, 71100 Kırıkkale, Turkey; halil.dasgin@gmail.com; 3 Faculty of Medicine, Hacettepe University, Sıhhiye, 06100 Ankara, Turkey; ilyas@hacettepe.edu.tr

**Keywords:** fetal programming, low quality protein, pregnancy, rats

## Abstract

Several studies have indicated the influence of a maternal low protein diet on the fetus. However, the effect of a maternal low quality protein diet on fetal growth and development is largely unknown. Wistar rats (11 weeks old) were mated and maintained on either a chow diet with 20% casein (*n* = 6) as the control group (C), or a low quality protein diet with 20% wheat gluten (*n* = 7) as the experimental group (WG) through gestation and lactation. Maternal body weights were similar in both groups throughout the study. Birth weights were not influenced by maternal diet and offspring body weights during lactation were similar between the groups. Offspring’s plasma amino acid profiles showed that plasma methionine, glutamine and lysine were significantly lower and aspartic acid, ornithine and glycine-proline were significantly higher in the WG. Plant based protein comprises an important part of protein intake in developing countries. It is well-known that these diets can be inadequate in terms of essential amino acids. The current study shows differential effects of a maternal low quality protein diet on the offspring’s plasma amino acids. Future studies will examine further aspects of the influence of maternal low quality protein diets on fetal growth and development.

## 1. Introduction

Proteins are essential macronutrients that form enzymes, hormones, structural components and immune system cells through stimulation of protein synthesis [1,2]. Amino acids are constituents of proteins and provide specific functions in metabolism according to their chemical properties [3]. Since animal and plant based foods differ in their essential amino acid contents and their digestibility, human diets may have different protein qualities [4,5]. High quality proteins are those that are easily digestible and include essential amino acids in adequate amounts [5]. Regional fact sheets have indicated that there are significant differences in intake of protein and essential amino acids between developed and developing countries [6]. It is well-established that developing economies’ nutrition is commonly based on cereals, grains and legumes which include insufficient digestible proteins and essential amino acids whilst developed economies nutrition is based on animal and vegetable sources that consist of high quality protein [7,8].

Maternal nutritional status is one of the factors that may influence long-term risk of disease development during adulthood [9]. Although retrospective studies have shown the link between size at birth and risk of cardiovascular diseases and type 2 diabetes later in life [10,11], the hypothesis of fetal programming has very limited data in humans in terms of ethics and multifactorial nature of human life [12]. To date several animal studies have examined the effects of low protein diets on the developing fetus and have shown that maternal low protein diets can be associated with the development of chronic diseases through altered metabolic parameters [13,14,15,16,17]. While there is extensive data on programming effects of maternal low protein diets on fetal development, the influence of protein quality on a possible similar programming capacity is unexplored. Understanding the impact of a maternal low quality protein diet is of major importance as several human studies have indicated that diets of different protein quality or amino acid content can affect appetite [18], lipemia [19,20], blood pressure [21] and insulin resistance [22].

It has been demonstrated that the quality of dietary protein is a crucial determinant of growth and metabolism in animal models [23,24,25]. Few studies have investigated the influence of low quality protein diets during non-gestational periods in rats and have revealed that a low quality protein diet, which includes wheat gluten as the source of protein, can be effective in inducing altered growth [26,27]. However, the effects of wheat gluten protein when compared to a high quality protein source in isocaloric quantities during gestation and lactation are unknown. Therefore, the aim of this study is to examine the effects of a maternal wheat gluten protein diet during gestation and lactation on maternal and fetal plasma amino acid concentrations, fetal growth and development until weaning.

## 2. Experimental Section

### 2.1. Animals and Diets

**Figure 1:**
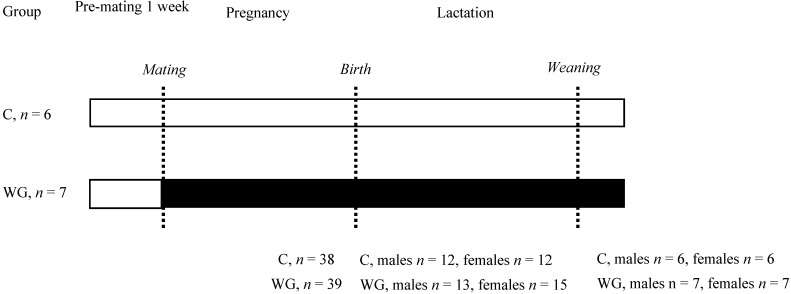
Study design. C, control diet; WG, wheat gluten diet. Values for *n* on the left show the number of successful pregnancies in each group and n at the bottom show the number of offspring that were used for analyses.

The experiments were performed under the license from the Ethics Committee of Hacettepe University, Ankara, Turkey, number: 2014/17. All animals were housed individually in plastic cages and subjected to a 12 h light-dark cycle at a temperature of 20–22 °C and 45% humidity. The animals were housed on shavings and had *ad libitum* access to food and water at all times. After one week of habituation period, Virgin female Wistar rats (aged 11 weeks) were paired with a Wistar stud male and mating was confirmed by the appearance of a semen plug. Then animals were randomly allocated to be fed either a control chow diet with 20% casein (C; *n* = 6) or an experimental diet with 20% wheat gluten (WG; *n* = 7) throughout gestation and lactation. Diets (MBD, Kocaeli, Turkey) were isocaloric and consisted of 20% protein, 4.1% fat and 75.9% carbohydrates. There was no supplementation of sulfur-containing amino acids in the diets. Both diet groups were weighed in and out of the cage daily. At birth, the birth weight and major organ weights of all litters were recorded. The litters were then culled to a maximum of four pups (two males and two females, where possible, randomly selected). At the end of lactation, mothers and one male and female offspring from each litter were culled using CO_2_ asphyxia and cervical dislocation after overnight fasting. Blood samples were taken by cardiac puncture, and major organs were weighed and snap-frozen in liquid N_2_. The remaining offspring were fed and were to be used in a different study. Figure 1 shows the study design. 

### 2.2. Estimation of Milk Yield

Milk yield was estimated by the method of Sampson and Jansen [28]. Average body weights between the third and thirteenth day of lactation and daily body weight gain of offspring were used in the following equation:

Yield = 0.0322 + 0.0667 (weight) + 0.877 (gain)

### 2.3. Plasma Amino Acid Concentration

All blood samples were collected into heparinized capillary tubes and stored on ice until centrifuged in a hematocrit centrifuge. Plasma was collected and stored at −80 °C until required for analysis. Plasma amino acid analyses were performed using a GC amino acid kit (EZ:faast, Phenomenex, Torrance, CA, USA) [29]. Maternal and offspring amino acid concentrations were analyzed in samples, which were taken at the end of lactation.

### 2.4. Statistical Analysis

All data were analyzed using the Statistical Package for Social Sciences (version 16; SPSS, Inc., Chicago, IL, USA). The effect of gestational diet on maternal and fetal outcomes was assessed using a general linear model analysis of variance (ANOVA) (fixed factors, maternal diet and sex). Where longitudinal data were available (for example, weekly body weights or energy intake), the week of study was used in a repeated-measures analysis. Values are expressed as mean values with their standard errors. *p* < 0.05 was considered statistically significant. No *post hoc* analyses were performed. The study was adequately powered to meet the stated aim.

## 3. Results

**Figure 2:**
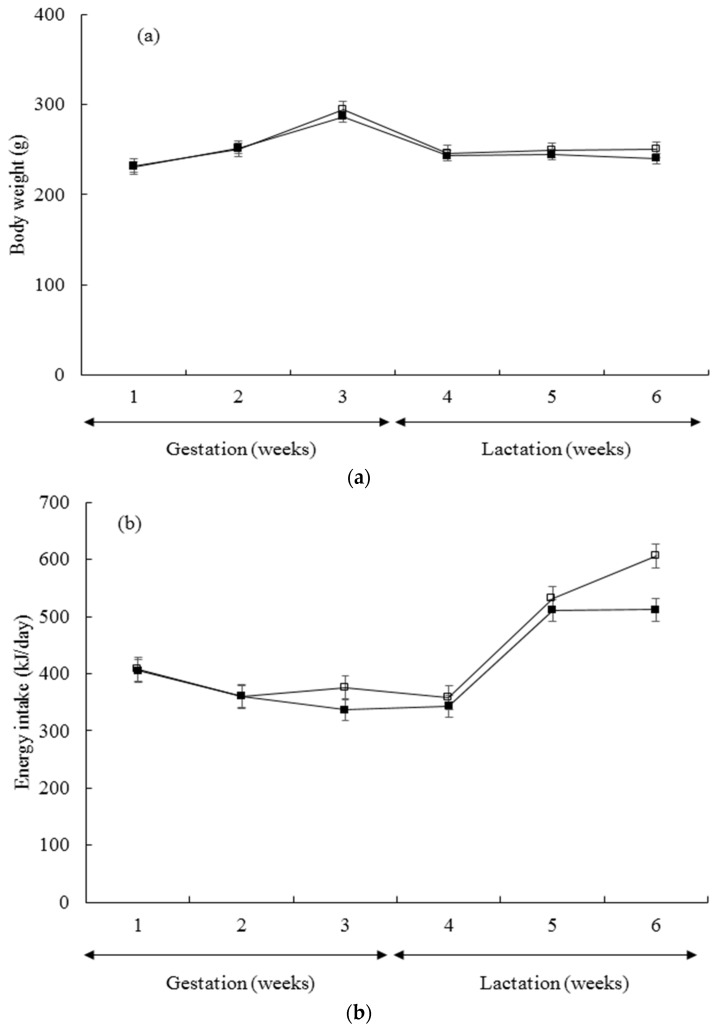

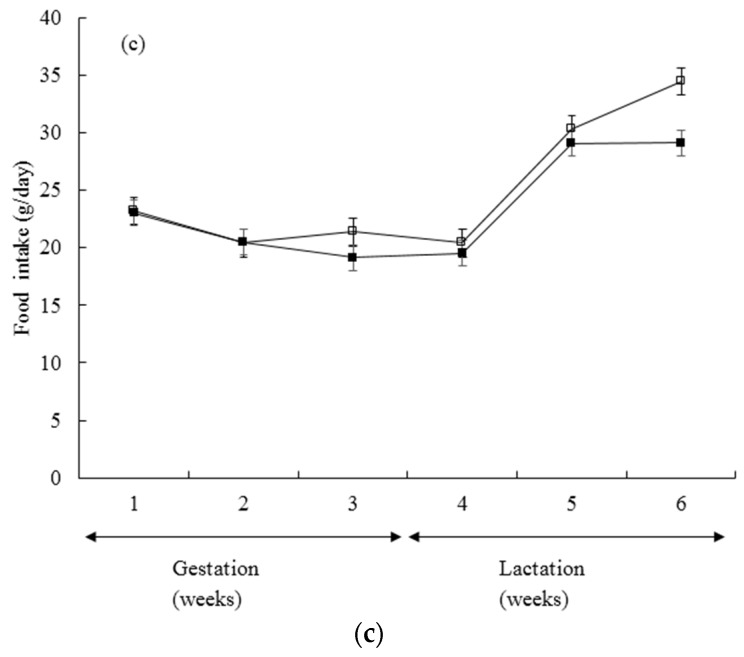
Values are means with standard errors represented by vertical bars. (**a**) Body weight changes during pregnancy and lactation in rats fed control (C, *n* = 6) or low quality protein (WG, *n* = 7) diets; (**b**) Average daily energy intakes during each week of pregnancy and lactation period in rats fed control (C, *n* = 6) or low quality protein (WG, *n* = 7) diets; (**c**) Average daily food intakes during each week of pregnancy and lactation periods in rats fed control (C, *n* = 6) or low quality protein (WG, *n* = 7) diets.

The body weights of the mothers did not vary significantly at the start of the experiment (C: 222.65 ± 5.14 g, WG: 220.91 ± 4.75). All animals became pregnant and only one rat failed to carry pregnancy to day 20 of gestation. Although study weeks significantly affected maternal body weights (*p* < 0.001), they were not influenced by diet throughout the study period as shown in Figure 2a. Litter size did not vary significantly between the groups and there was no effect of diet on overall litter size (C: 10.34 ± 1.42, WG: 9.57 ± 1.32). Figure 2b shows the energy intake of rats during the study period. Energy intake of WG group was significantly lower than C group (effect of diet, *p* = 0.017 and study weeks, *p* < 0.001). Similarly, food intake was significantly lower in WG when compared to C as seen in Figure 2c (effect of diet, *p* = 0.019 and study weeks, *p* < 0.001).

Low quality protein diet during gestation did not affect birth weight of offspring (Table 1), and this trend continued during lactation in both genders. No effect was observed except the effect of study week (*p* < 0.001, Table 1). Despite lower energy intake in mothers of the WG group, estimation of milk yield was similar between the groups (Table 1). Liver, brain, heart, left and right kidney weights of offspring, which were culled at birth and at the end of lactation, were measured (Table 2). WG offspring’s major organ’s weight remained similar to C.

Table 3 shows essential and non-essential maternal and offspring plasma amino acid concentrations at weaning. Overall, maternal amino acid concentrations exhibited similar results between C and WG groups. However, maternal serine was significantly lower in WG (*p* = 0.046). When the offspring’s amino acid concentrations were examined at the end of weaning, several amino acids were found to be altered in WG. Glutamine (*p* = 0.044), lysine (*p* = 0.033) and methionine (*p* = 0.05) were significantly reduced in WG offspring whereas aspartic acid (*p* = 0.005), glycyl-proline (*p* = 0.007) and ornithine (*p* = 0.05) were significantly increased (Table 3). In addition, there was a tendency towards significance in serine (*p* = 0.08) and hydroxyproline (*p* = 0.06), which indicates that these amino acids may be higher and lower in the WG group, respectively. Offspring/maternal amino acid ratio also revealed altered concentrations as a result of maternal diet. Offspring/maternal ratio of aspartic acid (*p* = 0.007), serine (*p* = 0.003), glycyl-proline (*p* = 0.023), valine (*p* = 0.007) and ornithine (*p* = 0.046) were significantly higher in WG whilst on the contrary, the offspring/maternal ratio of hydroxyproline (*p* = 0.037), lysine (*p* = 0.002) and methionine (*p* = 0.033) were significantly lower compared to C. Furthermore, there was a tendency towards significance in tyrosine (*p* = 0.074), threonine (*p* = 0.074) and asparagine (*p* = 0.081), which may suggest an enhanced ratio of offspring/maternal amino acid concentration in WG. 

**Table 1:** Birth weight, offspring’s body weight during lactation and estimation of milk yield.

	Males		Females	
	C	WG	C	WG
Birth weight (g)	5.96 ± 0.14	6.15 ± 0.14	5.53 ± 0.14	5.58 ± 0.13
Body weight at 1st week (g) *	11.41 ± 0.82	11.45 ± 0.79	10.71 ± 0.82	10.82 ± 0.73
Body weight at 2nd week (g) *	22.61 ± 0.82	22.55 ± 0.79	21.95 ± 0.82	22.03 ± 0.73
Body weight at 3rd week(g) *	35.83 ± 0.82	34.40 ± 0.79	34.80 ± 0.82	33.56 ± 0.73
Estimation of milk yield (g/day)	5.17 ± 0.39	4.95 ± 0.39	5.12 ± 0.39	5.63 ± 0.39

Values are means with standard errors. Birth weight data were analyzed in rats fed maternal control (C, males *n* = 36, females *n* = 36) or low quality protein (WG, males *n* = 39, females *n* = 45) diets. Body weight during lactation (1st, 2nd and 3rd weeks) were analyzed in rats fed a maternal control (C, males *n* = 12, females *n* = 12) or low quality protein (WG, males *n* = 13, females *n* = 15) diet. * Body weights were significantly influenced by study week (*p* < 0.001).

**Table 2:** Organ weight of offspring at birth and end of lactation.

Organ (% Body Weight)	At birth (Both Genders)		End of Lactation (Males)		End of Lactation (Females)	
	C	WG	C	WG	C	WG
Liver	3.47 ± 0.20	3.77 ± 0.19	3.61 ± 0.21	3.56 ± 0.19	3.81 ± 0.26	3.72 ± 0.24
Brain	2.92 ± 0.15	3.23 ± 0.14	3.08 ± 0.14	2.77 ± 0.13	3.29 ± 0.14	3.28 ± 0.13
Heart	0.54 ± 0.03	0.52 ± 0.02	0.40 ± 0.05	0.36 ± 0.04	0.43 ± 0.05	0.35 ± 0.05
Left kidney	0.48 ± 0.28	0.49 ± 0.03	0.63 ± 0.03	0.66 ± 0.02	0.64 ± 0.03	0.67 ± 0.02
Right kidney	0.49 ± 0.28	0.51 ± 0.03	0.65 ± 0.03	0.69 ± 0.02	0.66 ± 0.03	0.70 ± 0.03

Values are means with standard errors. C, control chow diet; WG, low quality protein diet. Organ weight data at birth were analyzed in rats fed maternal control (C, males *n* = 36, females *n* = 36) or low quality protein (WG, males *n* = 39, females *n* = 45) diets. Organ weight data at the end of lactation were analyzed in rats fed a maternal control (C, males *n* = 6, females *n* = 6) or low quality protein (WG, males *n* = 7, females *n* = 7) diet.

**Table 3:** Maternal and offspring’s plasma amino acid concentrations at weaning.

Amino Acid (µmol/L)	Maternal		Offspring		Offspring /Maternal	
Essential amino acids	**C**	**WG**	**C**	**WG**	**C**	**WG**
Lysine *^,§^	25.67 ± 4.57	34.70 ± 4.23	177.90 ± 41.96	38.11 ± 38.85	8.65 ± 1.34	1.25 ± 1.25
Phenylalanine	859.12 ± 54.98	759.60 ± 50.90	688.99 ± 59.07	638.89 ± 54.69	0.90 ± 0.13	0.88 ± 0.12
Leucine	307.73 ± 23.02	259.49 ± 21.31	267.55 ± 25.51	261.76 ± 23.62	0.91 ± 0.07	1.02 ± 0.06
Isoleucine	237.55 ± 18.73	203.53 ± 17.34	243.11 ± 19.19	224.45 ± 17.76	1.04 ± 0.07	1.12 ± 0.06
Methionine *^,‡^	90.15 ± 6.60	74.20 ± 6.11	493.59 ± 117.86	183.88 ± 109.12	7.62 ± 1.54	2.51 ± 1.42
Valine ^§^	337.05 ± 23.43	287.98 ± 21.69	130.89 ± 57.56	257.86 ± 53.29	0.23 ± 0.15	0.89 ± 0.14
Histidine	1700.26 ± 151.67	1499.39 ± 140.42	1347.24 ± 167.45	1483.52 ± 155.03	0.82 ± 0.14	1.06 ± 0.13
Threonine	414.30 ± 38.45	363.86 ± 35.60	335.35 ± 31.12	398.02 ± 28.81	0.89 ± 0.09	1.12 ± 0.08
Non-essential amino acids						
Proline	292.34 ± 19.67	258.47 ± 18.21	423.57 ± 65.52	447.99 ± 60.66	1.59 ± 0.18	1.70 ± 0.16
Hydroxyproline ^‡^	38.84 ± 3.00	34.20 ± 2.78	70.83 ± 10.74	43.94 ± 9.94	2.27 ± 0.31	1.27 ± 0.29
Glycyl-proline **^,‡^	129.90 ± 16.41	121.75 ± 15.19	96.65 ± 29.32	227.54 ± 27.15	0.67 ± 0.39	2.09 ± 0.36
Serine ^†,§^	465.83 ± 31.84	368.11 ± 29.48	352.42 ± 40.34	458.58 ± 37.34	0.81 ± 0.09	1.25 ± 0.08
Glycine	658.13 ± 74.92	603.96 ± 69.36	1068.50 ± 90.78	1144.75 ± 84.05	1.70 ± 0.20	1.98 ± 0.19
Aspartic acid **^,§^	495.58 ± 48.37	452.08 ± 44.78	136.09 ± 56.83	419.29 ± 51.88	0.28 ± 0.13	0.90 ± 0.12
Asparagine	138.77 ± 8.84	115.44 ± 8.18	136.21 ± 6.83	131.00 ± 6.32	1.02 ± 0.05	1.16 ± 0.05
Cystathionine	56.85 ± 31.22	87.07 ± 28.90	76.37 ± 26.77	137.37 ± 21.86	9.66 ± 3.88	4.70 ± 3.17
Ornithine *^,‡^	594.09 ± 195.16	438.89 ± 180.69	416.47 ± 235.44	1104.42 ± 217.97	0.79 ± 1.82	6.35 ± 1.68
Tyrosine	833.19 ± 83.97	767.22 ± 77.74	716.87 ± 124.31	909.47 ± 115.08	0.94 ± 0.09	1.18 ± 0.08
Cystine	71.34 ± 15.37	59.99 ± 14.23	75.20 ± 12.11	68.70 ± 11.21	1.21 ± 0.44	1.62 ± 0.41
Alanine	956.86 ± 93.76	891.79 ± 86.80	704.67 ± 109.46	850.89 ± 101.34	0.81 ± 0.09	0.98 ± 0.08
Sarcosine	11.95 ± 0.72	12.52 ± 0.61	20.06 ± 1.42	20.04 ± 1.31	1.75 ± 0.18	1.63 ± 0.15
Glutamic acid	706.61 ± 46.32	646.88 ± 42.88	724.82 ± 66.08	748.66 ± 61.18	1.06 ± 0.05	1.15 ± 0.05
Glutamine *	244.59 ± 109.55	283.26 ± 92.59	332.48 ± 83.39	73.72 ± 77.21	30.50 ± 12.85	9.80 ± 10.86

Values are means with standard errors. C, control chow diet; WG, low quality protein diet. Maternal plasma amino acid concentrations were analyzed in rats fed control (C, *n* = 6) or low quality protein (WG, *n* = 7) diets. Offspring’s plasma amino acid concentrations were analyzed in rats fed maternal control (C, males *n* = 6, females *n* = 6) or low quality protein (WG, males *n* = 7, females *n* = 7) diets. ^†^ Maternal mean value was significantly different from that of the C group (*p* < 0.05). * Offspring mean value was significantly different from that of C group (*p* < 0.05). ** Offspring mean value was significantly different from that of the C group (*p* < 0.001). ^‡^ Offspring/maternal ratio mean value was significantly different from that of the C group (*p* < 0.05). ^§^ Offspring/maternal ratio mean value was significantly different from that of C group (*p* < 0.001).

## 4. Discussion

The influence of a maternal low quality protein diet on the developing offspring is unknown in contrast to the more direct inferences of maternal and neonatal health. Few animal studies support the notion that low quality protein diets with wheat gluten may trigger differences in metabolic functions and physiology [26,27,30,31]. However, it is not clear whether such effects can continue on throughout pregnancy and lactation and subsequently affect the health status of offspring. The primary aim of this preliminary study was to compare the effect of a maternal low quality protein diet with wheat gluten to that of a high quality protein diet with casein in terms of fetal development and amino acid concentrations. The current study successfully demonstrated that there were differential effects of the maternal low quality protein diet on the plasma amino acid concentrations of the offspring in an animal model.

It has previously been shown that the amino acid balance may impact food intake and body weight regulation in rats [32,33,34]. A study, which investigated the effect of protein quality and quantity during lactation, reported that maternal food intake was significantly lower in a wheat gluten group than in a group receiving casein [35]. Similar results were obtained from another study with the implication of a significantly decreased food intake during lactation but not during gestation [36]. In the current study, a significant decrease of 6.49% occurred in the WG group’s overall energy intake during gestation and lactation combined, and this effect appeared to initiate at the end of the second week of gestation. The decline in voluntary food intake as a result of low quality protein diet feeding can be explained by alterations in the central nervous system, since the central nervous system and histamine receptors have been shown to be affected by dietary protein quality manipulations [37]. Despite having a lower energy intake, WG’s maternal body weights did not differ when compared to C. As the amount of protein in the WG diet was not reduced and animals were fed with an isocaloric diet, the 6.49% reduction may not have been sufficient to induce a significant change in body weight during the 6-week study period.

There is a large body of evidence indicating that birth weight can be a potential predictor of programming effects in animal models [38,39]. Some studies reported a decreased birth weight following maternal low quality protein diets [40,41] whereas a different study showed no effect of 21% wheat gluten [42]. Correspondingly, the current study did not observe any difference in birth weight of offspring or as body weights during lactation. These results are consistent with other aspects of the study since maternal milk yield estimation and offspring’s organ weights also did not differ between the groups. Hence, it can be suggested that a 20% wheat gluten diet does not exert a significant effect upon growth and development of offspring at weaning.

The manipulative effect of dietary protein quality and quantity on maternal serum and organ amino acid composition has been shown previously [35,42]. In these studies a common conclusion stated that with an improvement in protein quality or quantity, levels of most essential amino acids increased in the serum and other tissues such as the brain and mammary glands. In the current study, a 20% wheat gluten diet did not exert a profound effect on maternal plasma amino acid levels. This can be attributable to the period of sample withdrawal, which was at the end of lactation, and levels of circulating amino acids during early or late gestation, and early lactation are of interest. Nevertheless, serine was significantly lower in mothers that had the WG diet. Serine is considered as an important amino acid due to its primary endogenous methyl donor role in the carbon metabolism [43]. It has been shown that deficiency of maternal methyl donors during pregnancy and lactation can be responsible for altered epigenetics, metabolism and cognitive function in offspring [44,45]. More specifically, histotroph, which provides additional nutrients in the form of uterine secretions to developing offspring, includes serine along with other amino acids such as methionine [46]. Therefore, inadequate maternal serine can be a risk factor for long-term consequences for the health of the fetus. Another important point is that due to decreased energy intake, there was also a reduced protein intake in the WG group. When highly digestible casein protein is compared to less digestible wheat gluten, both quantity and quality of the protein in the WG group may lead to differences in availability of amino acids. 

This is the first study comparing the influence of dietary protein quality on offspring amino acids within the field of fetal programming. It was assumed that the amino acid composition of casein and wheat gluten would be the key factors in determining the metabolic fate of the ingested amino acids in the offspring. When the amino acid compositions of the two protein sources are compared, it appears that aspartic acid, threonine, serin, alanine, valine, methionine, isoleucine, leucine, tyrosine and histidine are lower in wheat gluten with the limiting amino acid lysine [47]. For lysine and methionine, this was reflected in the plasma concentrations, as these amino acids were found to be significantly lower in WG offspring. Despite a lack of an altered growth pattern in these animals, it is interesting to observe these differences in amino acid levels, because in previous work it have been shown that different dietary lysine levels might influence growth rates of rats up to 80% [48]. However, this effect appears to be dependent on the degree of restriction since a study showed that a severe restriction of 75% was associated with growth retardation while a moderate restriction of 50% did not affect growth rate [49]. Similarly, a diet lacking methionine (0%) was linked to growth retardation in mice [50]. Therefore, the level of restriction of amino acids may have a deterministic effect on growth and development. However, these studies did not involve the lactating period. Protein modifications take place on lysine residues during post-translational stages [51,52]. This combined with insufficient methionine can lead to the suggestion that having lower concentrations of these amino acids during neonatal life may affect future developmental processes and long-term health. 

The two amino acids aspartic acid and glutamine did not exhibit a corresponding concentration in the plasma compared to the amino acid compositions of the two protein sources. Despite a lower content in wheat gluten, offspring’s aspartic acid concentrations showed a significant increase in the plasma, and despite a higher content in wheat gluten, offspring’s glutamine showed a significant decrease. Although more data is required to explain these findings, the adaptive nature of the fetal metabolism may contribute to an altered utilization of glutamine and aspartic acid in the metabolism. For instance, glutamine is one of the well supplied and most used amino acids in fetal life during late gestation [53] and, therefore, it may be over utilized in replacement of other limiting amino acids in the fetal metabolism. In addition, arginine and glutamine were shown to be linked with altered muscle growth and decreased protein synthesis during late pregnancy in a different animal model [54]. As the programming of muscle development by protein restriction has been shown in several pervious studies [55,56], these outcomes can be examined in protein quality studies.

In general, offspring’s plasma concentrations of many amino acids are greater than those found in the maternal plasma [57]. With respect to offspring/maternal amino acid ratio data in the current study, this situation was observed in most amino acids. Despite a slight change in maternal plasma amino acid concentrations, offspring/maternal amino acid ratios indicated a number of significant alterations in WG when compared to C. Taken together, offspring/maternal ratios of methionine, lysine, ornithine, glycyl-proline and aspartic acid in the WG group exhibited different results when compared to C group. Therefore, further studies should evaluate the metabolisms of these amino acids. 

One of the limitations of this study is the lack of nitrogen balance data. In order to determine the exact effect of these parameters on maternal and offspring metabolism, nitrogen balance measurements are required in future studies. In addition, measuring the observed and suggested parameters during early gestation is necessary to elucidate the exact influences. Further, the diets in the current study were not supplemented with sulfur amino acids, as one study, which investigated the effects of protein quality and quantity during lactation did not report a profound influence on protein scores with 22.3% casein without supplementation [35] and others indicated that 20% casein was sufficient to induce the required growth parameters [26,27,58]. However, casein supplemented with l-cystine is recommended to induce a maximum rate of growth in rodents [59]. Therefore, the lack of growth and plasma cysteine differences between the groups can also be attributable to the non-supplementation of sulfur amino acids in the casein diet. It is worth noting that, despite a lack of sulfur amino acid supplementation in C, plasma methionine levels of the WG offspring were still considerably lower. Consequently, the outcomes of this preliminary study need to be examined in future studies in terms of modified dietary amino acid compositions of different low quality protein diets, early and late gestational metabolism and phenotype of offspring.

## 5. Conclusions

Dietary protein quality is still an issue in under-developed and developing countries. Deficiencies due to essential amino acid intakes in pregnant women’s diets may influence pregnancy outcomes and future health of offspring. In the current study, for the first time consumption of a maternal low quality protein diet exerted differences on plasma amino acid concentrations of weaning rats. Growth and development of offspring did not appear to be influenced by a 20% wheat gluten diet at weaning. Since the ongoing effects of these differences on adulthood of the offspring is not known, the translation of these differences to metabolic consequences and the resulting phenotype need to be investigated in further studies.
